# Bacterial Concentration Detection using a PCB-based Contactless Conductivity Sensor

**DOI:** 10.3390/mi10010055

**Published:** 2019-01-14

**Authors:** Xiao-Yan Zhang, Zhe-Yu Li, Yu Zhang, Xiao-Qian Zang, Kosei Ueno, Hiroaki Misawa, Kai Sun

**Affiliations:** 1 State Key Laboratory of Urban Water Resource and Environment, Harbin Institute of Technology, Harbin 150090, China; xyzhang774985529@163.com (X.-Y.Z.); zhylee@hit.edu.cn (Z.-Y.L.); yuzhang429@126.com (Y.Z.); 1112710105@hit.edu.cn (X.-Q.Z.); 2 Research Institute for Electronic Science, Hokkaido University, Sapporo 001-0021, Japan; k-ueno@es.hokudai.ac.jp (K.U.); misawa@es.hokudai.ac.jp (H.M.); 3 Department of Applied Chemistry & Institute of Molecular Science, National Chiao Tung University, Hsinchu 30010, Taiwan

**Keywords:** bacterial concentration, capacitively coupled contactless conductivity detection (C^4^D), capillary, *E. coli*, printed-circuit-board (PCB)

## Abstract

Capacitively coupled contactless conductivity detection (C^4^D) is an improved approach to avoid the problems of labor-intensive, time-consuming and insufficient accuracy of plate count as well as the high-cost apparatus of flow cytometry (FCM) in bacterial counting. This article describes a novel electrode-integrated printed-circuit-board (PCB)-based C^4^D device, which supports the simple and safe exchange of capillaries and improves the sensitivity and repeatability of the contactless detection. Furthermore, no syringe pump is needed in the detection, it reduces the system size, and, more importantly, avoids the effect on the bacteria due to high pressure. The recovered bacteria after C^4^D detection at excitation of 25 Vpp and 60–120 kHz were analyzed by flow cytometry, and a survival rate higher than 96% was given. It was verified that C^4^D detection did not influence the bacterial viability. Moreover, bacteria concentrations from 10^6^ cells/mL to 10^8^ cells/mL were measured in a linear range, and relative standard deviation (RSD) is below 0.2%. In addition, the effects on bacteria and C^4^D from background solutions were discussed. In contrast to common methods used in most laboratories, this method may provide a simple solution to in situ detection of bacterial cultures.

## 1. Introduction

Over the past decades, bacteria have been widely used in many fields, including pharmacy [1], chemistry [2], biology [3], environmental science [4], and fermentation [5]. Bacterial counting, as one of the most important indicators, is used to determine the concentration of bacterial culture, to monitor the water quality, to assess pollution level, and to diagnose patients. Although there are many techniques developed to count bacteria, such as plate count and optical density (OD), these techniques are mostly time-consuming, labor-intensive, and unable to provide sufficient accuracy [6]. Some methods based on fluorescent dyes, for example, fluorescence analysis, and flow cytometry (FCM), are advanced in rapidness, technical simplicity, and efficiency, even in distinguishing different physiological states of bacteria at single cell level [7,8,9]. Nevertheless, fluorescent quenching and high cost per test [10] are still limiting it from being widely used. Biosensors have been paid more and more attention with the development of micro electro mechanical system (MEMS) [11], which provide a portable and real-time platform [12,13]. In recent years, by making use of MEMS point-of-care testing, technologies have become an effective and rapid method to detect bacteria through analyzing the image [14,15,16,17,18]. Photoelectrochemical and electrochemical biosensors have been widely used for bioanalytical proposes [19], detecting bacterial strains [20], capturing bacteria [21], and monitoring activity of bacterial fermentation [22,23]. Electrochemical detection has a rapid response speed [24] and has the potential to be a useful tool in low-concentration applications [25,26]. However, this method cannot identify those microbes that rely on specific antibodies [27,28,29,30]. In addition, electrodes used in electrochemical detection are easily contaminated by the sample, which influences the results. Surface Enhanced Raman Scattering (SERS) avoid these shortcomings, and it can detect microorganisms without specific antibodies [31,32,33]; however, the weak signal is still a problem to be solved.

Capacitively coupled contactless conductivity detection (C^4^D), by analyzing and testing the conductivity change of sample [34], could simply and sensitively detect metal cations, amino acids, and organic ions in beer, wine, milk, potable water, and juice [35,36,37,38]. The electrodes of C^4^D contain three electrodes, the excitation electrode to which AC signal is applied, the shield electrode, and the pick-up electrode, which measured the detection signal. Two metal needles, with capillary passing through, were used as excitation electrode and pick-up electrode in early C^4^D devices; at the same time, to improve the sensitivity of detection, silver conductive adhesive was used to narrow the gap between capillary and needle. To improve the signal-to-noise ratio (S/N) and integration, a printed-circuit-board (PCB) based C^4^D with shorter electrical connection of the electrodes to the current/voltage operational amplifier was introduced to connect capillaries and microfluidic chips [39,40,41]. Silva et al. integrated the circuit and the wire-wrapped electrodes on a 18 × 18 mm^2^ PCB, and optimized amplitude and frequency for each column diameter and electrophoretic buffer [42,43]. Jaanus et al. also integrated electrodes on the PCB and improve detection sensitivity by using an idle capillary for compensation [44]. 

The application of C^4^D was mainly for detecting ions, while there are still some researchers focusing on counting cell by it. Emaminejad et al. [45] was the first to count cells from sheet whole blood based on contactless conductivity. Chen et al. [46] succeeded in counting 9-μm MCF-7 and 15-μm HCM cancer cells on a C^4^D integrated chip. They both achieved label-free counting. Although electrical impedance spectroscopy has been used for detection of bacteria counting, there have been few studies focusing on the bacterial counting by C^4^D [47]. This is because bacteria are smaller organisms with single cell volume in the range of 0.1–1 µm^3^/cell, which is only one tenth of a cell. To detect bacteria by C^4^D is much more difficulty comparing to the cells. 

In this paper, we developed a type of novel C^4^D device with both electrodes and amplifiers integrated on the PCB. Copper via holes of PCB with 400-µm inner diameter were used as C^4^D electrodes through which a 360-μm OD capillary passes exactly. The fabrication of the via holes on PCB could provide the inner-diameter (ID) of 300-μm minimum with the tolerance less than 50 μm, which confirms better accuracy and repeatability. The bacterial suspension was dragged into the capillary manually by a syringe, without a syringe pump. The detection is very fast because of the concentration detection mechanisms instead of peak counting. Although the proposed method is not able to distinguish different populations of bacteria, this is a simple, inexpensive, rapid, contactless, and label-free method that enables bacterial counting without damage to the cells.

## 2. Materials and Methods 

### 2.1. Materials and Reagents

Standard broth was used as a bacterial growth medium, and phosphate-buffered saline (PBS, pH = 7.4, NaCl 8.0 g/L, KCl 0.2 g/L, Na_2_HPO_4_ 1.42 g/L, KH_2_PO_4_ 0.24 g/L) was prepared in the laboratory. SYBR Green I (Invitrogen, Eugene, OR, USA) was diluted in dimethylsulfoxide (DMSO) to a ratio of 1:100 and then stored at −20 °C prior to use. Propidium iodide (PI) and albumin from bovine serum (BSA) was from Sigma (shanghai, China) and was stored at 4 °C. All samples were incubated in 1 mg/mL PI for 15 min in the dark before flow cytometry (BD, Becton, Dickinson and Company, Franklin Lakes, NJ, USA) measurement. Glutaraldehyde, paraformaldehyde, ethyl alcohol, and isoamyl acetate were from TCI (Tokyo, Japan). All dilutions were carried out in deionized water from a Millipore system.

### 2.2. Bacterial Culture and Sample Preparation

**Figure 1:**
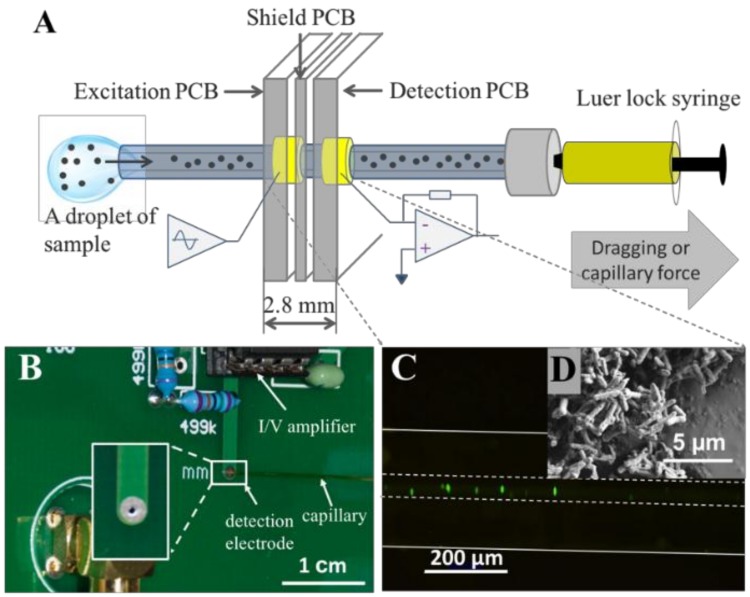
(**A**) Schematic illustration of capacitively coupled contactless conductivity detection (C^4^D) device on detecting bacterial concentration. (**B**) The printed-circuit-board (PCB) based electrodes. (**C**) Fluorescence-labeled *Escherichia coli* in capillary. (**D**) Scanning electron microscope (SEM) image of *E. coli*, the length of which on average is about 2 μm.

*Escherichia coli* (CGMCC 1.2385, China General Microbiological Culture Collection Center, Beijing, China) was cultured in the broth medium at 37 °C for 17 h in an incubator to achieve 10^7^ cells/mL. The bacteria were then centrifuged at 4000 rpm for 5 min to remove the broth medium and were re-suspended in PBS followed by centrifuging again. Prior to the experiment, the capillary was flushed with 20 mg/L BSA solution to avoid the non-specific cell adhesion to the surface. To ensure that the bacteria was successfully injected into the capillary, which passed through the detection cell (Figure 1A), a concentration of 1.0 × 10^7^ cells/mL sample was stained by SYBR Green I and was observed under the microscope with a charge-coupled device (CCD) camera (IX-73x microscope and DP80 camera, Olympus, Japan). The polyimide coating of the capillary was peeled off to eliminate fluorescent interference before observation. It can be seen clearly that the stained bacteria with green fluorescence pass through, and there was no aggregation found in the capillary (Figure 1B). 

### 2.3. E.coli Preparation for SEM

Moreover, *E. coli* was characterized by SEM (Figure 1D). It shows that the *E. coli* is approximately 2–3 μm long and 0.5 μm wide. *E. coli* preparation for SEM observation includes the flowing steps. After incubation in nutrient broth for 17 h at 37 °C, *E. coli* was washed by PBS three times to remove the broth and then soaked in 4% glutaraldehyde for 2 h, immersed in 3% paraformaldehyde for 1 h, successively dehydrated in 30%, 50%, 75%, and 80% ethyl alcohol for 10 min and in 95% for 20 min, stored in isoamyl acetate for 30 min, and dried out overnight. The samples should be washed by PBS three times in each step and deposited onto a clean silicon wafer. 

### 2.4. Bacteria Loading 

For C^4^D detection, different concentrations of the samples were loaded into 200-μL tubes and were then injected into a 150-μm-ID and 10-cm-long capillary (Yongnian Optic Fiber Plant, Yongnian, Hebei, China) for 5 s by pulling back a syringe manually instead of a syringe pump. The outlet diameter of untreated polyimide coated fused silicon capillary is 360 μm. The output voltage signal was then recorded after stopping the injection. This rapid injection method could avoid the contamination from the syringe and the Luer lock. Furthermore, pump-free injection could avoid the destruction of the sample and conductivity changing due to high pressure in the capillary from the pump’s pushing.

## 3. Results and Discussion

### 3.1. Design Strategy and Instrumentation

The experimental setup comprised a home-made C^4^D and a data acquisition system. A block diagram of the C^4^D circuitry, which contains an excitation PCB, a detection PCB, and a shielding PCB, is given in Figure 1A. Two solder pads, working as an excitation electrode and pick-up electrode, were located in the corresponding PCBs. The ID of the electrodes was 400 µm, which was decided by the 360-μm OD of the capillary used in the following experiments. The width of both the excitation and detection electrodes was 1.0 mm which was decided by the thickness of the excitation and detection PCBs. The thickness of the shielding PCB is 0.8 mm, which served as the gap between the two electrodes. Hence, the detection cell size was 2.8 mm (shown in Figure 1A). The capillary was washed with deionized (DI) water, PBS, and BSA in turn prior to use, then passed the excitation, shielding, and detection PCBs in sequence. The three PCBs were assembled with quite accurate alignment holes. The shielding PCB could effectively lower the noise and could control the gap between two electrodes, which could avoid the shortcut problem. Hence, the above design supported a smooth, safe, and simple exchange of capillaries and improved the sensitivity and repeatability of the contactless detection. A field programmable gate array (FPGA) was used to generate a sine-wave excitation signal with the designed frequency. The sinusoidal signal with 100 kHz frequency and 25 Vpp (peak-to-peak) amplitude was applied to the excitation electrode after optimization. The pick-up amplifier consisted of two cheap (unction Field-Effect Transistor) JFET-input operational amplifiers (LF-357, Texas Instruments, Austin, TX, USA) as the transimpedance amplifier and voltage follower. The signal was then rectified with a lock-in synchronous amplifier (AD630, Analog Devices, Norwood, MA, USA), two-order Chebyshev low-pass filtered, and programmable gain amplified (PGA205, Burr-Brown, Austin, TX, USA). The output was finally fed via a coaxial cable to a 16-bit data acquisition system (NI 6229, National Instruments, Austin, TX, USA). The system was controlled under LabVIEW software. Figure 1B shows the photograph of the prototype C^4^D system. The signal-to-noise (S/N) of this system was detected by the different concentrations of the potassium chloride solution from 1 to 10 μM (Figure S1). The detection at the level of 1 μM is illustrated, and a linear relationship (R^2^ = 0.9885) was obtained at this low concentration. Figure S2 shows the detection limit of 0.1 μM potassium salt solution, the S/N of which is higher than 9.

### 3.2. Electrical Effect of C^4^D on Bacteria Viability

Bacteria can be killed when placed to voltage pulses of high strength for sufficient time, thus, there is a risk of bacteria damage or viability affection by the electrical detection. Especially for some rare uncultured bacteria, which are difficult to culture because of their complex growing conditions and long growing period, the risk may lead to unexpected loss. Hence, it is very important to discuss the bacterial viability before and after detection [48]. C^4^D is an electrical detection method that measures conductivity corresponding to bacteria concentrations; thus, the influence of voltage amplitude and frequency of the wave on bacteria viability will be discussed. Wang et al. reported that the cell membrane would be disintegrated by the stimulation over 1000 V/cm of the electric field strength, which resulted in nearly 100% cell death; however, low field strength less than 300 V/cm did not affect the cell viability in the exposure period of 30–40 s [49,50]. In addition, the membrane potential induced by an external field is another factor need to be considered in detection. The membrane potential of bacteria is the difference in and out of cell. The critical valve is 1.1 Vm for bacteria to be in stationary growth phase [51]. The permeabiltization of cell membrane would increase when the external field was applied, which would lead to bacterial lysis and death. In our C^4^D system, the measured current through the media in the capillary was less than 1 μA at the excitation of 25 Vpp, though the voltage was larger than the one used in electric double layer [52,53,54]. The maximum conductivity of the media was 0.286 mS/cm measured by METTLER TOLEDOLE 740 (METTLER TOLEDO, Shanghai, China), that is, the resistivity is 416.7 Ω·cm. The length of the C^4^D cell is 2.8 mm (shown in Figure 1A); and the diameter of C^4^D cell is 150 μm, which is the ID of capillary. Hence, the electric field strength in the capillary was about 2.36 V/cm, which is far below 300 V/cm. According to the theoretical equation [51], the membrane potential of 0.288 mV for *E. coli* cell at the excitation of 25 Vpp is revealed. It is far below the critical membrane potential of 1.1 Vm, which is only 0.1% of the Vm.

**Figure 2:**
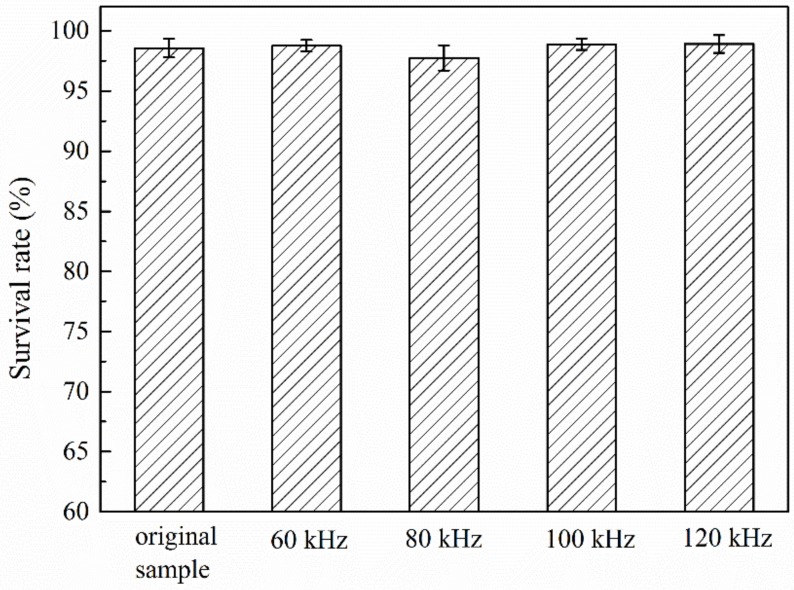
Influence on bacteria viability due to C^4^D detection by analyzing the ratio of live to dead cells. The recovered bacteria were labeled with Propidium Iodide (PI) and analyzed by flow cytometry (FCM). Error bars are standard deviations of four measurements.

Frequency is another electrical parameter that will influence the bacterial viability. Shawki [55] recently reported that the current with low frequency less than 100 Hz (about 10 V/cm, 130 s exposure) can be used as a physical method to kill bacteria, with a death rate over 40%, however, bacteria exposure to higher frequencies showed insignificant lethal effect; the death rate at 100 kHz was less than 1%. 100 kHz is the working frequency of C^4^D. In our assessment experiment, the *E. coli* samples with the concentration of 10^7^ cells/mL in PBS buffer were exposed in the capillary with external sinusoidal excitation of 25 Vpp and 60–120 kHz. The viability of bacteria before and after conductivity detection was analyzed using FCM after being fluorescent-labeled by Propidium Iodide (PI). This essay is a commonly used method to analyze the cell viability [56]. PI is unable to pass through the membrane of live bacteria, thus, the damaged bacteria will be labeled by PI. Figure 2 shows that the survival rate after exposure at the frequencies of 60 kHz, 80 kHz, 100 kHz, and 120 kHz were all above 96%, with very small error bars. It can be concluded that the working frequency in range from 60–120 kHz of C^4^D may not influence the viability of the bacteria obviously. Additionally, numerous studies have provided evidence that alternating current (AC) frequency of 5 kHz–10 MHz [57] can been used in the application of dielectrophoretic cell separation. Cells could be successfully cultured after dielectrophoretic separation. 

### 3.3. Optimization of PBS Concentration for Bacteria Counting 

The conductivity of the membrane of live bacteria is only ~10^−3^ μS/cm, thus, bacterial membrane is highly insulated [58]. The conductivity of bacteria’s interior can be as high as ~10 μS/cm, which is much higher than 0.055 μS/cm of DI water. Thus, when live bacteria are suspended in DI water, ions will release from the *E. coli* and generate osmotic shock. At the same time, water will enter into the bacteria, and cause death of most bacteria due to the bacterial membrane broken [47]. Therefore, PBS buffer is widely used for substance solution and cell container rinsing, which is able to balance pH for better bacterial viability and provide same ion concentration. Electrical impedance spectroscopy makes use of this characteristic to detect bacteria; however, the electrodes are easily contaminated and corroded due to direct sample contacting [47].

**Figure 3:**
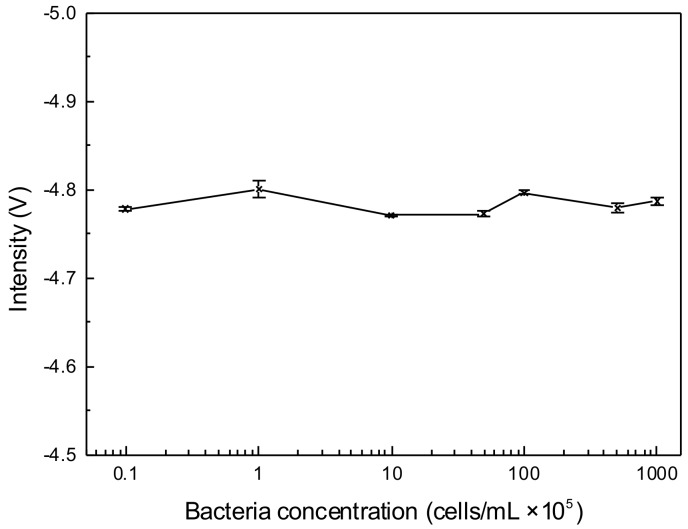
The C^4^D response to the bacteria suspended in original phosphate-buffered saline (PBS) buffer with concentrations from 10^4^ to 10^8^ cells/mL. Error bars are standard deviations of four measurements.

**Figure 4:**
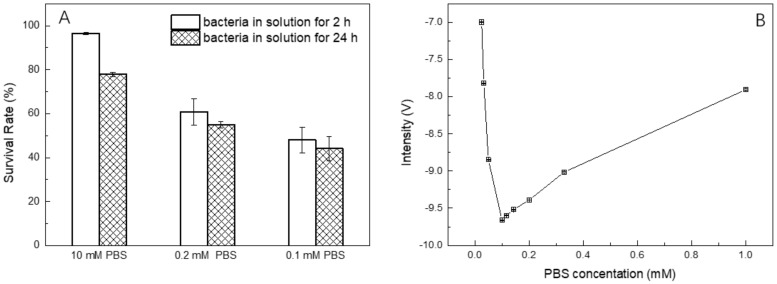
(**A**) Bacteria survival rate in 10 mM PBS solution, 0.2 mM PBS dilution, 0.1 mM PBS dilution and water after 2 h and 24 h were detected by FCM. Bacteria were dyed by PI for 10 min. Error bars are standard deviations of 3 measurements. (**B**) The relationship between PBS dilution and C4D intensity. Error bars are standard deviations of 4 measurements.

The osmolality and ion concentrations of PBS solutions are equal to those of bacteria. However, because the conductivity of PBS buffer is also roughly the same as the bacterial interior, it is hard to detect the conductivity change after suspending the bacteria with the concentration from 10^4^ cells/mL to 10^8^ cells/mL in the 10 mM PBS buffer (see Figure 3). Hence, it is essential to reduce the conductivity of background solution. It was reported that bacteria could survive in the solution, in which the concentration of sodium chloride is higher than the critical value of 1.7 mM [59]. To understand the effect of salt concentration on the bacteria, the bacteria survival rate in the original PBS buffer and different diluted PBS buffer were measured by FCM. Figure 4A shows that the survival rate of bacteria in 10 mM PBS buffer for 2 h was 96%, and decreased with the descent of the PBS concentration. Variation between 2 h and 24 h detection for 0.2 mM and 0.1 mM PBS dilution was less than 10%.

The 10 mM PBS buffer was then diluted to different concentrations to measure corresponding C4D intensities (shown in Figure 4B). It is noted that the C4D intensities indicate a negative correlation in case of the PBS concentration higher than 0.1 mM dilution, however, a strong positive correlation from 0.1 mM to 0.025 mM. It could be explained that in the case of low conductivity detection the C4D impedance is essentially a function of the capillary inner solution conductivity, while at high conductivities, impedance is mainly determined by capacitances of the capillary wall [60]. Low concentrations, less than 0.1 mM PBS solution, exhibit a high quality of linearity, whereas the ion concentration of sodium chloride is much lower than the critical value. Hence, we finally selected 0.2 mM and 0.1 mM PBS dilution as C4D background for discussion.

**Figure 5:**
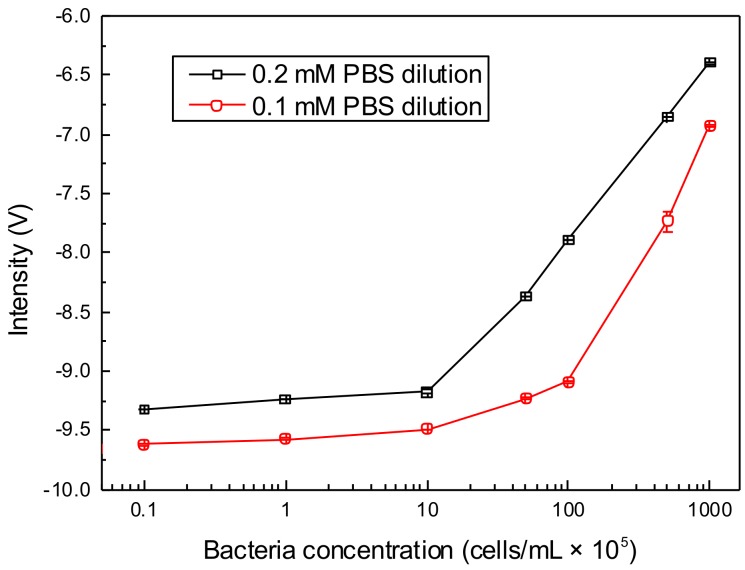
The C^4^D response to the bacteria suspended in 0.2 mM PBS dilution and 0.1 mM PBS dilution with the different concentrations of 10^4^–10^8^ cells/mL. Error bars are standard deviations of four measurements.

Figure 5 shows the relationship between the C^4^D intensity and different bacteria concentrations from 10^4^ to 10^8^ cells/mL in 0.2 mM and 0.1 mM PBS dilutions. In both PBS dilutions, C^4^D intensities increase with the increase of bacteria concentration, and give higher sensitivities when detecting bacteria in the range of 10^6^–10^8^ cells/mL in contrast with the concentration less than 10^6^ cells/mL. It seems that the detection in 0.2 mM PBS indicates a little better response than that in 0.1 mM PBS in the case of low bacterial concentration. The S/N of 10^4^ and 10^5^ cells/mL bacteria in 0.2 mM PBS is 55.7 and 124.3, respectively.

## 4. Conclusions

In summary, we designed and fabricated a novel C^4^D device to count *E. coli*. The electrodes and the circuits were integrated on three PCBs. Thus, the design supports a simple and safe change of capillaries to improve the sensitivity and repeatability comparing to traditional C^4^D methods. Firstly, we discussed the electric field strength and the frequency effect on the bacteria after the C^4^D detection; results indicate that the survival rate is above 96% at the excitation of 25 Vpp and 100 kHz. Secondly, the background solutions for C^4^D detection of bacteria were discussed. For the in situ detection of bacteria culture, we suggest 0.2 mM PBS solution as the C^4^D background, which adapts to detecting the bacteria concentration in the range of 10^6^–10^8^ cells/mL. In addition, the consumption solution is nanoliter scale, and no syringe pump is needed, which avoids the high pressure effect on the bacteria. Hence, this method may provide a good technical solution for many applications and the comparison of developed devices and this work is in Table A1. Reasonable direction for further research is to improve the detection range by increasing the ID of capillary, which can enlarge the volume of detection cells. In addition, narrow-ID-capillary array or holey fibers can be used to improve the detection limit by lower the background from the PBS buffer.

## Supplementary Materials

The following are available online at http://www.mdpi.com/2072-666X/10/1/55/s1, Figure S1: The C^4^D detection of different concentrations of the potassium chloride solution from 1 to 10 μM (R^2^ = 0.9885), Figure S2: Response to the injection of 0.1 μM KCl solution. The flow velocity is 3 μL/min of DI water, and the injection volume of sample is 1 nL.

## Appendix A

**Table A1:** The comparison of developed devices and this work.

Method	Operation	Recording	Label	Accuracy	Reference
Optical density (OD)	simple	automatic monitoring	no	providing the growth trend	[6]
Plate count	simple	manual reading	no	depending on labor	[6]
Flow cytometry	skilled operator needed	automatic monitoring	fluorescence	good	[7,8,9]
This work	simple	automatic monitoring	no	Better than OD in range of 10^6^–10^8^ cells/ml	
