# Supplementary material for: Bacterial Concentration Detection using a PCB-based Contactless Conductivity Sensor
Source: Micromachines (Basel). 2019 Jan 14;10(1):55. doi: 10.3390/mi10010055 (PMC6356519; doi:10.3390/mi10010055)
Supplement: Supplementary file 1 [file micromachines-10-00055-s001.pdf]

Supplementary Materials: Bacterial Concentration Detection using a PCB-based Contactless Conductivity Sensor

Xiao-Yan Zhang, Zhe-Yu Li, Yu Zhang, Xiao-Qian Zang, Kosei Ueno, Hiroaki Misawa and Kai Sun

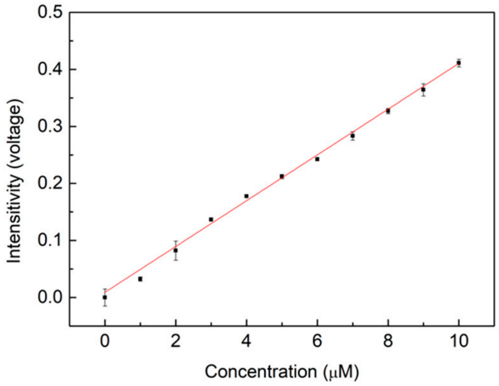

Figure S1. The C^4D detection of different concentrations of the potassium chloride solution from 1 to 10 μM (R2 = 0.9885).

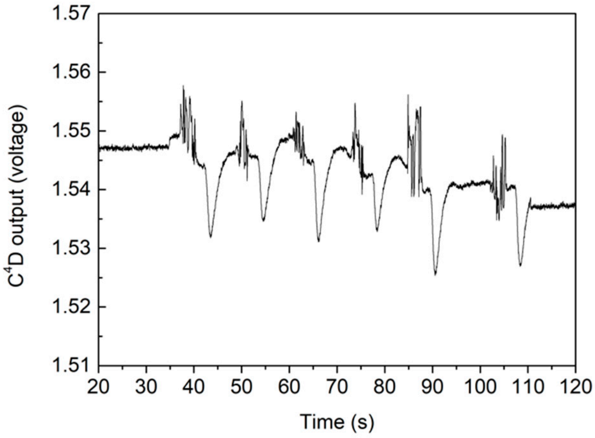

Figure S2. Response to the injection of 0.1 μM KCl solution. The flow velocity is 3 μL/min of DI water, and the injection volume of sample is 1 nL.
